# Carriage of Carbapenem-Resistant Enterobacterales in Adult Patients Admitted to a University Hospital in Italy

**DOI:** 10.3390/antibiotics10010061

**Published:** 2021-01-10

**Authors:** Pamela Barbadoro, Daniela Bencardino, Elisa Carloni, Enrica Omiccioli, Elisa Ponzio, Rebecca Micheletti, Giorgia Acquaviva, Aurora Luciani, Annamaria Masucci, Antonella Pocognoli, Francesca Orecchioni, Marcello Mario D’Errico, Mauro Magnani, Francesca Andreoni

**Affiliations:** 1 Department of Biomedical Science and Public Health, Università Politecnica delle Marche, 60122 Ancona, Italy; p.barbadoro@staff.univpm.it (P.B.); e.ponzio@staff.univpm.it (E.P.); rebeccamicheletti@libero.it (R.M.); g.acquaviva@pm.univpm.it (G.A.); a.luciani@pm.univpm.it (A.L.); m.m.derrico@staff.univpm.it (M.M.D.); 2 SOD Igiene Ospedaliera-AOU Ancona Associated Hospitals, 60126 Ancona, Italy; 3 Department of Biomolecular Sciences, University of Urbino, 61029 Fano, Italy; daniela.bencardino@uniurb.it (D.B.); mauro.magnani@uniurb.it (M.M.); 4 Diatheva srl, 61030 Cartoceto, Italy; e.carloni@diatheva.com (E.C.); e.omiccioli@diatheva.com (E.O.); 5 SOS Microbiologia Laboratorio Analisi, AOU Ancona Associated Hospitals, 60126 Ancona, Italy; Annamaria.Masucci@ospedaliriuniti.marche.it (A.M.); Antonella.Pocognoli@ospedaliriuniti.marche.it (A.P.); Francesca.Orecchioni@ospedaliriuniti.marche.it (F.O.)

**Keywords:** Enterobacteriaceae, PCR-based replicon typing, antibiotic-resistance, sequence types, multilocus sequence typing, plasmids

## Abstract

The emerging spread of carbapenemase-producing Enterobacterales (CPE) strains, in particular, *Klebsiella pneumoniae* and *Escherichia coli*, has become a significant threat to hospitalized patients. Carbapenemase genes are frequently located on plasmids than can be exchanged among clonal strains, increasing the antibiotic resistance rate. The aim of this study was to determine the prevalence of CPE in patients upon their admission and to analyze selected associated factors. An investigation of the antibiotic resistance and genetic features of circulating CPE was carried out. Phenotypic tests and molecular typing were performed on 48 carbapenemase-producing strains of *K. pneumoniae* and *E. coli* collected from rectal swabs of adult patients. Carbapenem-resistance was confirmed by PCR detection of resistance genes. All strains were analyzed by PCR-based replicon typing (PBRT) and multilocus sequence typing (MLST) was performed on a representative isolate of each PBRT profile. More than 50% of the strains were found to be multidrug-resistant, and the *bla*_KPC_ gene was detected in all the isolates with the exception of an *E. coli* strain. A multireplicon status was observed, and the most prevalent profile was FIIK, FIB KQ (33%). MLST analysis revealed the prevalence of sequence type 512 (ST512). This study highlights the importance of screening patients upon their admission to limit the spread of CRE in hospitals.

## 1. Introduction

The rapid spread of carbapenem-resistant Enterobacterales (CRE) mediated by carbapenemase enzymes represents a serious problem in hospitals worldwide [1]. Carbapenemases are beta-lactamases that have the ability to hydrolyze penicillins, cephalosporins, monobactams and carbapenems rendering them ineffective as antibiotics [2]. The use of broad-spectrum antimicrobials is a risk factor for the colonization of CRE in healthcare settings, and the lack of alternative therapies increases the mortality and morbidity rates as well as the costs of prolonged hospitalizations. Currently, the European epidemiology of CRE is variable. It is endemic in some countries, such as Italy, Greece and Romania, whereas its spread is still limited in most other European countries, notwithstanding a growing incidence in Spain, Portugal and Bulgaria [3].

The frequent exchange of plasmids carrying carbapenemase genes occurring among the strains increases the risk of CRE infections [4]. The European Antibiotic Surveillance Network (EARS-Net) data for 2018 reported frequent cases of carbapenem-resistance, in particular, related to the spread of carbapenemases-producing *Klebsiella pneumoniae* (KPC-Kp) and *Escherichia coli* (CP-Ec), with higher levels associated with the former. An increasing incidence of carbapenem-resistance in the EU/EEA population was reported between 2015 and 2018, with a growing number of deaths caused by *K. pneumoniae* infections. Despite its limited incidence, the distribution of *E. coli* resistant to carbapenems also needs to be monitored, considering the global impact of antimicrobial resistance [5]. In Italy, CRE have been spreading since 2010, and recent national surveillances data show that 95% of carbapenem resistance is attributable to *K. pneumoniae* and *E. coli* isolated from bacteraemia.

The main clinical characteristics and risk factors associated with CRE colonization of vulnerable patients include comorbidities, recurrent hospitalization, lengthy hospitalization and complex therapeutic management [6]. However, the seriousness of CRE carriage may vary according to each patient’s particular clinical situation. For example, it is considered serious in patients with complicated intra-abdominal and urinary tract infections that require long hospital stays [3]. As suggested by the European Centre for Disease Prevention and Control (ECDC 2018) [5], a range of hygiene control measures must be implemented in healthcare settings. Moreover, in light of the importance of the role of patient transfers in CPE spread in the healthcare network, such measures must be supported by the systematic screening of patients upon their admission and during their hospitalization [7].

The identification of CPE carriage through the active rectal surveillance of patients is an effective way to limit and control CPE spread in healthcare settings. When setting up a CPE surveillance program, relevant considerations include the program’s level of automation, its costs, the time it requires to execute, as well as how easy it is to use. Rapid molecular methods may be advisable to support screening performed in clinical microbiology laboratories [3].

The investigation described herein aims to determine the prevalence of CRE in patients upon their admission to a teaching hospital in Central Italy and to analyze selected associated factors. Moreover, a characterization of the strains was performed using PCR-based replicon typing (PBRT), PCR resistance genes and multilocus sequence typing.

## 2. Results

### 2.1. Antimicrobial Resistance

In the study period, 2478 patients were screened on admission by rectal swab. Overall 48 tested positive upon their first admission; hence, the prevalence of CRE was 1.93%. The isolated strains were predominantly *K. pneumoniae* (94%, *n* = 45), while the detection of *E. coli* was infrequent (*n* = 3). All strains were carbapenem-resistant and a phenotypic test revealed a resistance mediated by KPC carbapenemase enzymes in 47 strains, classifying them as carbapenemase-producing Enterobacterales (CPE). Genotypic characterization of the main carbapenemase-resistance genes showed that all strains harbored the *bla*_KPC_ resistance gene with the exception of an *E. coli* strain; *bla*_KPC_ was combined with *bla*_VIM_ in a single strain of *K. pneumoniae*. Twenty-one isolates (44%) were classified as multidrug-resistant (MDR) as indicated by Magiorakos et al. [8]. Only seven isolates, identified as *K. pneumoniae*, were susceptible to all of the tested antibiotics. Moreover, the highest resistance rate was towards cefuroxime (54%) followed by ciprofloxacin (44%), levofloxacin (42%) and ampicillin sulbactam (42%). The genotypic and phenotypic patterns of antibiotic-resistance and PBRT profiles of the isolates are summarized in Table 1.

### 2.2. Plasmid Typing and Classification

**Figure 1:**
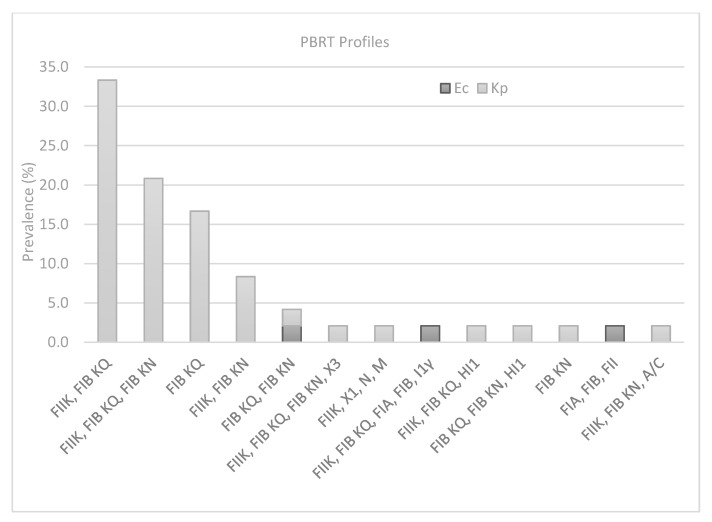
Prevalence of PBRT profiles in *K. pneumoniae* (Kp) and *E. coli* (Ec) strains.

Plasmid analysis showed that IncFIB KQ was the most predominant Incompatibility group observed in 81% of the strains. The combined results obtained from PBRT identified 14 profiles with a prevalence of FIIK, FIB KQ (33.3%; *n* = 16), FIB KQ (16.6%; *n* = 8), FIIK, FIB KQ, FIB KN (20.8%; *n* = 10), FIIK, FIB KN (8.3%; *n* = 4), whereas a single strain was found to be positive for the remaining profiles (Figure 1). Multireplicon status (two or more replicons) was recorded in 39 strains (81%), with a maximum of five replicons in one of the three strains of *E. coli,* which also showed resistance to six antibiotics. By contrast, the *K. pneumoniae* strain (reported in Table 1 as 245) showed a high number of replicons (*n* = 4) but low resistance. The only strain (654 in Table 1) carrying a combination of two carbapenem-resistance genes *bla*_KPC_; *bla*_VIM_ was found among the most prevalent PBRT profile (FIIK, FIB KQ). Finally, FII was the only replicon recorded for the *E. coli* strain, which was the only strain found to be negative for carbapenemase-resistance genes.

### 2.3. Multilocus Sequence Typing Analysis

MLST analysis was performed on a representative isolate from each PBRT profile, and based on these results, 14 strains (11 *K. pneumoniae* and 3 *E. coli* strains) were characterized as shown in Table 1. Five different Sequence types (STs) were identified: ST512, ST101, ST405, ST307 and ST131. Specifically, among the 11 strains of *K. pneumoniae,* three STs were determined with a prevalence of ST512 (*n* = 5), followed by ST101 (*n* = 4) and ST307 (*n* = 2). All the STs detected in this study were found in strains showing high variability in terms of both antimicrobial resistance and PBRT patterns. The strains called Kp_506 and Kp_176, belonging to ST512, were susceptible to all of the tested antibiotics and associated with the FIB KQ and the FIIK, FIB KQ, FIB KN group, respectively. In addition, the Kp_245 strain was resistant to a single antibiotic (AMS) but associated with a multireplicon status showing four replicons (FIIK, FIB KQ, FIB KN, X3). By contrast, the MDR strain Kp_6 was resistant to five antibiotics (gentamycin (CN), amikacin (AK), tobramycin (TOB), ciprofloxacin (CIP), cefuroxime (CXM)) but only two replicons were detected (FIIK, FIB KQ). Finally, Kp_15, resistant to three antibiotics (CN, CIP, CXM), was the only strain showing a FIIK, FIB KQ, HI1 profile. The same variability was observed for ST10, which is shown in Table 1. Importantly, the two strains of *K. pneumoniae* classified as ST307 showed a high level of resistance with different antibiotic patterns.

On the other hand, two STs were detected among the three strains of *E. coli*: the well-known ST131 (*n* = 1) and the sporadic ST405 (*n* = 2). The former was associated with a strain showing a high resistance rate and five replicons, whereas the latter was associated with two strains showing different resistance patterns and PBRT profiles. In addition, one of those strains was negative for carbapenem-resistance genes, confirming the variability of the collection.

### 2.4. Epidemiological Data

The control patients did not differ from the cases in terms of age, sex, hospital admission rate during the previous 30 days, or comorbidities. However, there was a significant difference between the two groups in terms of origin upon admission, with 83.3% of the controls coming from the emergency department (ED) versus 52.1% of the cases. Moreover, none of the controls had previously resided in a long-term care facility (LTCF) (Table 2).

The results of multivariable logistic regression are shown in Table 3, highlighting a significant association between previous antibiotic use (OR 3.76, 95%CI 1.45–9.72) and hospital admission (OR 3.00, 95%CI 1.16–7.71) and CPE carriage on admission. On the other hand, admission to the ED was protective (OR 0.27, 95%CI 1.10–0.73).

## 3. Discussion

In Italy, the rapid spread of CRE has become endemic, and it is a critical issue in the surveillance and treatment of infections [9].

CRE infection control and prevention require greater investments than other diseases across a range of areas, including patient screening, the management of long hospitalizations, as well as antimicrobials and patient isolation [3]. Once they are introduced by newly admitted patients, CRE strains rapidly spread through the hospital; hence, the prompt identification of colonized patients through active rectal surveillance can potentially reduce transmission. Incorporating rapid inexpensive methods into the clinical routine of hospitals to typify the plasmid conferring carbapenem-resistance can help to stem the spread of Enterobacterales [10].

We confirmed the efficacy of active screening for CRE through rectal swabs as an important component of any infection control program [11]. The observed high prevalence of *K. pneumoniae* was in agreement with other investigations, including the last European Survey [5,12]. Most of our CRE isolates were found to be resistant to many antibiotics commonly used in hospital settings, a phenomenon that has been widely reported [9,13,14]. These findings highlight the limited number of therapies that are available to treat CRE, which accounts for the high mortality rate currently associated with this type of infection [12]. Hence, the control of the spread of CRE infections is critically important, particularly in hospital settings.

Among the known groups of genes encoding for the carbapenemase enzymes, *bla*_KPC_ and *bla*_NDM_ are the most prevalent, and the co-occurrence of multiple resistance determinants is frequently reported [15,16]. However, in the present study, only the variant *bla*_KPC_ was detected in the isolates, and only one strain of *K. pneumoniae* was positive for two carbapenemase genes. To our knowledge, such a low incidence of the co-occurrence of *bla*_KPC_ and *bla*_VIM_ has rarely been described [17,18].

Considering the global spread of these determinants through plasmids, the characterization of incompatibility groups among CPE isolates makes it possible to track the dissemination of plasmids where antibiotic resistance genes can be located [16]. Not surprisingly, the most prevalent incompatibility group detected among our isolates was IncF, the common plasmid types largely associated with the spread of antibiotic resistance genes in Enterobacterales [19]. This is due to their advantageous intracellular adaptation supported by the regulatory sequences of replicons in constant and rapid evolution. Furthermore, the IncF are low-copy-number plasmids carrying more replicons to promote the initiation of replication. This feature was also observed in the present work, in which a high number of multiple replicons were detected with a prevalence of FII and FIB, typically found in a multireplicon status. Normally, the FII replicon is silent, whereas the activity of FIB, as well as that of FIA, is only related to enteric bacteria. Notably, the occurrence of multiple replicons allows plasmids to enlarge the host range replication, increasing the likelihood that they may be transferred between different species [20]. Our analysis thus highlights the usefulness of molecular typing to better understand the distribution of resistant strains in clinical settings monitored by surveillance programs. Furthermore, the proposed method for the amplicon analysis using the AATI Fragment Analyzer reduces detection time and simplifies the electrophoresis step with an automated workflow. The PBRT method therefore represents a valid tool that can also be used in hospital settings thanks to its rapidity.

The identification of ST through MLST allowed us to collect preliminary information about the clones circulating in the hospital. Of all of the sequence types obtained, ST512 and ST131 were the most frequent in *K. pneumoniae* and *E. coli*, respectively. ST512 belongs to the clonal complex 258 (CC258), in which ST258 is not only the dominant type but also the ancestor of all members. This finding confirms the endemic nature of ST512 in Italy, its association with several plasmids containing *bla*_KPC_ variants and its widespread rectal colonization [21,22]. In addition to the endemic ST512 clone, *K. pneumoniae* ST101 was also identified, and found to carry *bla*_KPC_, pointing to the high potential for the spread of this clone, which is already found in Italy [23]. Notably, a comparative analysis carried out by Roe and colleagues (2019) revealed a similar resistome between emerging ST101 and the global ST258 of *K. pneumoniae*, strengthening the tendency of the former to become an epidemic dual-risk clone [24]. In agreement with other studies carried out in Italy, emerging ST307 was also identified among our isolates, confirming the pivotal role of this clone in clinical niches [25,26]. It is a novel distinctive lineage carrying KPC-plasmids acquired through horizontal transfer with particular virulence factors allowing an advantageous adaptation to the hospital environment [26].

Notwithstanding the limited number of *E. coli* strains collected, two relevant lineages were detected. ST405 has an international distribution and is associated with extended-spectrum beta-lactamases [27]. ST405, carrying *bla*_NDM_, is usually responsible for urosepsis [28]; however, in our study this gene was found to be negative. Commonly, the acquisition of novel resistance determinants is essential for a successful and rapid spread of emerging clones. ST131 causes a wide range of extraintestinal infections with a high prevalence in patients in LTCF, who require regular health-care assistance [29]. The majority of the plasmids associated with ST131 belong to the IncF group containing FIA and FII replicons as was observed in the present study. The IncF plasmid carries multiple antibiotic determinants and virulence factors that ensure important advantages for ST131 throughout host colonization [21,29].

The identification of clones responsible for the spread of resistance determinants as well as the detection of emerging clones highlight the evolutionary dynamics of bacterial strains. Recent studies have identified the ED as an important reservoir for CRE colonization, highlighting the need to address infection control in the ED in order to better manage carbapenem resistance in other wards [30,31]. Our results, on the other hand, show entrance from ED as a protective factor. This discrepancy can probably be accounted for by the fact that in the present investigation, patients originating from the ED were admitted through that department but did not stay there for a prolonged period. In addition to being an established factor associated with colonization, previous admission to a LTCF was not independently associated with CPE carriage. However, we must underscore the role of LTCFs as a reservoir of CPE in the healthcare system and the specific clones belonging to patients originating from LTCF [32]. In this context, the patient’s disability and need for assistance highlight the importance of contact precautions and infection control policies. The continuous collection of epidemiological and molecular information in healthcare facilities allows such facilities to enact prompt intervention to mitigate the risk of the colonization of patients during their stay.

## 4. Materials and Methods

### 4.1. Bacterial Isolates

A surveillance study involving the collection of isolates provided by the ongoing CRE screening on admission program at Ancona Associated Hospitals (Marche Region, Italy) was carried out from February to September 2018. A total of 2478 patients were screened on admission by rectal swab in the study period.

Rectal swabs from all adult patients were collected on admission to the hospital at the clinical microbiology laboratory in Ancona (Ancona Associated Hospitals, Italy). All samples were analyzed for the presence of carbapenem-resistant bacteria. The rectal swabs were transported to our laboratory on the day of collection and stored at 4 °C until processed. The swabs were then inoculated into 5 mL Tryptic Soy Broth (Liofilchem, Roseto degli Abruzzi, Italy) with a 10 µg meropenem disc (Becton Dickinson, Franklin Lakes, NJ, USA) and incubated overnight at 35 °C [33]. Subsequently, 100 µL of a 0.5 McFarland suspension of each swab sample were inoculated into chromogenic media (Brilliance CRE Agar-Thermo Fisher, Waltham, MA, USA). After 24 h of incubation at 37 °C, the plates were evaluated to verify the color of the colonies: pale pink colonies were considered presumptive carbapenem-resistant *E. coli*, while steel-blue colonies were assumed to be *K. pneumoniae*. Colorless or cream-colored colonies were assumed to be *Acinetobacter baumannii*. Subsequently, VITEK MS (bioMérieux, Marcy l’Etoile, France) was used to confirm the species.

The phenotypic assay for carbapenem resistance mechanisms was carried out using disk diffusion method with meropenem disks supplemented with phenylboronic acid, dipicolinic acid and cloxacillin according to the instruction manual (Biolife Italiana, Milan, Italy) [34]. 

### 4.2. Antimicrobial Susceptibility Testing

Antimicrobial resistance patterns were identified by the Molecular Epidemiology Laboratory at the Università Politecnica delle Marche, Ancona (Italy). The antibiotic susceptibility of all the collected strains was determined by the minimal inhibitory concentration (MIC) method using the SensiQuattro Gram-negative System (Liofilchem, Italy). This methodology is in accordance with the guidelines of the European Committee on Antimicrobial Susceptibility Testing (EUCAST) clinical breakpoint version 9.0 (https://www.eucast.org/fileadmin/src/media/PDFs/EUCAST_files/Breakpoint_tables/v_9.0_Breakpoint_Tables.pdf). The following antimicrobial agents were tested: gentamycin (CN), amikacin (AK), tobramycin (TOB), piperacillin/tazobactam (TZP), fosfomycin (FOS), ampicillin-sulbactam (AMS), ciprofloxacin (CIP), levofloxacin (LEV), cefuroxime (CXM), co-trimoxazole (SXT).

### 4.3. Molecular Detection of Resistance Determinants

All carbapenem-resistant strains were tested for the detection of associated determinants. DNA was obtained by boiling the lysis of isolated colonies for 10 min in distilled water. The samples were then centrifuged at 15,000× *g* for 10 min and the supernatant was transferred into a new 1.5 mL tube and used for the following reactions. Carbapenemase-resistance genes (*bla*_IMP_, *bla*_VIM_, *bla*_OXA-48_, *bla*_NDM_, *bla*_KPC_) were determined by PCR amplification using primers and conditions previously described by Poirel et al. [35]. Briefly, the thermal cycling settings were 30 cycles at 95 °C for 30 s, 55 °C for 1 min, 72 °C for 30 s and one cycle at 72 °C for 5 min. A positive control was used for the amplification of each gene. The PCR products were separated by electrophoresis on a 2.5% (*w*/*v*) agarose gel (Sigma-Aldrich, St. Louis, MO, USA) and visualized using UV transillumination.

### 4.4. PCR-Based Replicon Typing

Plasmid characterization was performed by PBRT [10] using the PBRT kit 2.0 (Diatheva, Fano, Italy). This system, consisting of eight multiplex PCR assays, allows the identification of the following 30 replicons found in the Enterobacteriaceae family: HI1, HI2, I1, I2, X1, X2, X3, X4, L, M, N, FIA, FIB, FIC, FII, FIIS, FIIK, FIB KN, FIB KQ, W, Y, P1, A/C, T, K, U, R, B/O, HIB-M and FIB-M. All PCR reactions were performed according to the manufacturer’s instructions, including positive controls. The amplicons were detected through capillary electrophoresis on the AATI Fragment Analyzer (Agilent, Santa Clara, CA, USA) using the dsDNA 906 Reagent kit (Advanced Analytical, Ankeny, IA, USA). This amplicon analysis allows the combination of two multiplex PCRs in the same lane, resolving up to eight peaks. One µL of multiplex PCR 1 (M1) was combined with 1 µL of multiplex PCR 3 (M3), followed by M2 and M7, M6 and M8; the remaining M4 and M5 were loaded separately (2 µL each). The positive peaks were analyzed using the “PBRT plugin” developed in cooperation with the Advanced Analytical Company. This tool allows automatic peak calling and the recording of positive replicons.

### 4.5. Multilocus Sequence Typing (MLST)

MLST was performed on a representative strain for each PBRT pattern for both *K. pneumoniae* and *E. coli* strains. For the former, the amplification of the seven targeted housekeeping genes (*gapA*, *infB*, *mdh*, *pgi*, *phoE*, *rpoB*, and *tonB*) was carried out according to Protocol 2 of the MLST Institute Pasteur database (https://bigsdb.pasteur.fr/). This protocol uses primers with universal sequencing tails amplifying all genes at the same temperature and sequencing them with the same forward and reverse primers. Instead, MLST analysis for *E. coli* strains was performed using primers and conditions described by Wirth and colleagues [36] to detect the seven housekeeping genes *adk*, *icd*, *mdh*, *gyrB*, *purA*, *recA* and *fumC*. The same PCR primers were used for the sequencing carried out using the BigDye Terminator v. 1.1 Cycle Sequencing kit on the ABI PRISM^®^ 310 Genetic Analyzer (Thermo Fisher Scientific, Waltham, MA, USA). For each locus, the obtained consensus sequences were submitted to the Pasteur online database and compared to assign the specific allele numbers. Based on the seven-allele combination found for each locus, the profile of the isolates corresponding to a specific sequence type (ST) was determined.

### 4.6. Epidemiological Analysis

A case control approach was used to assess factors associated with the isolation of resistant strains upon hospital admission. CPE carriage was defined as colonization by a strain with a confirmed carbapenem-resistance phenotype. A 1:1 matched case-control study adjusted for sex and age (five-year range) was used. It included 48 cases and 48 controls. A control was defined as a patient not carrying CPE on admission. The following factors were investigated through the analysis of hospital discharge records, including socio-demographic variables (age, sex), provenance on admission (home, other healthcare institution, LTCF, ED), comorbidities (diabetes, renal disease, cardiovascular disease, chronic obstructive pulmonary disease (COPD), cancer, the presence of a disability, respiratory failure, urinary tract infection). All variables were tested by bivariate analysis for their association with CPE carriage on admission, and a multivariate regression model was constructed to evaluate variables independently associated with CPE carriage using a stepwise approach. The goodness of fit of the model was evaluated with the Hosmer-Lemeshow test and its discriminative ability was assessed with the area under the ROC curve. The level of significance was set at *p* < 0.05. Data were analyzed by Stata 15 software (Stata Corp).

Ethical approval was granted by the Regional Ethics Committee of the Marches (Det. 816/DG, 11 October 2018); in accordance with the study protocol, the clinical isolates were collected and stored as a routine clinical procedure, and information concerning both the clinical isolates and patient records was anonymous. All patients provided written informed consent for their data to be used for surveillance and preventive purposes, and anonymized data were thus linked by means of an ID given to each patient at the time of hospital admission.

## 5. Conclusions

In conclusion, our study highlights the importance of screening patients upon their admission in order to limit the spread of CRE in hospitals. Moreover, this study provides a proof of concept for the introduction of rapid molecular typing methods such as the PBRT in routine clinical microbiological screening. We tested the feasibility of this approach for future epidemiological surveillance programs and believe that it could be a good starting point to screen patients on admission and could enhance infection control/screening programs in hospitals.

## Figures and Tables

**Table 1:** Genotypic and phenotypic profiles of strains isolated from rectal swabs.

^a^ Isolate	^b^ PBRT Profile	^c^ CR Genes	^d^ Antibiotic Resistance										^e^ ST
			CN	AK	TOB	TZP	FOS	AMS	CIP	LEV	CXM	SXT	
Kp_6	FIIK, FIB KQ	*bla*_KPC_	R	R	R	S	S	S	R	S	R	S	512
Kp_14		*bla*_KPC_	S	S	S	S	S	S	S	S	R	S	
Kp_48		*bla*_KPC_	S	S	S	S	S	S	S	R	R	S	
Kp_56		*bla*_KPC_	S	S	S	S	S	S	S	R	R	S	
Kp_58		*bla*_KPC_	S	S	S	S	R	S	R	R	R	S	
Kp_194		*bla*_KPC_	S	S	S	S	R	S	S	S	R	S	
Kp_282		*bla*_KPC_	S	S	S	S	R	S	S	S	S	S	
Kp_484		*bla*_KPC_	R	R	R	S	S	S	R	R	R	R	
Kp_485		*bla*_KPC_	S	S	S	S	S	S	S	S	S	S	
Kp_593		*bla*_KPC_	S	S	R	R	R	R	S	S	R	S	
Kp_605		*bla*_KPC_	S	S	S	S	R	S	R	R	S	S	
Kp_612		*bla*_KPC_	R	R	R	S	S	S	R	R	R	R	
Kp_613		*bla*_KPC_	S	S	S	S	R	R	R	R	R	S	
Kp_654		*bla*_KPC_, *bla*_VIM_	S	R	R	S	R	R	R	R	R	R	
Kp_660		*bla*_KPC_	S	S	S	S	S	S	S	S	S	S	
Kp_672		*bla*_KPC_	R	R	R	S	S	S	R	S	R	S	
Kp_506	FIB KQ	*bla*_KPC_	S	S	S	S	S	S	S	S	S	S	512
Kp_604		*bla*_KPC_	S	R	R	S	S	S	R	R	R	S	
Kp_689		*bla*_KPC_	S	S	S	R	R	R	R	R	S	S	
Kp_690		*bla*_KPC_	S	S	S	R	R	S	S	S	S	S	
Kp_691		*bla*_KPC_	S	S	S	S	S	S	R	R	S	S	
Kp_696		*bla*_KPC_	S	S	S	R	S	S	S	S	S	S	
Kp_712		*bla*_KPC_	S	S	S	S	R	R	R	S	S	S	
Kp_714		*bla*_KPC_	R	R	R	S	S	S	S	S	S	S	
Kp_176	FIIK, FIB KQ, FIB KN	*bla*_KPC_	S	S	S	S	S	S	S	S	S	S	512
Kp_13		*bla*_KPC_	R	S	S	R	R	R	R	R	R	S	
Kp_59		*bla*_KPC_	S	S	S	R	R	R	S	S	S	S	
Kp_60		*bla*_KPC_	S	S	S	S	R	R	S	S	R	S	
Kp_186		*bla*_KPC_	S	S	S	S	S	S	S	S	S	S	
Kp_187		*bla*_KPC_	S	S	S	S	S	R	S	S	R	S	
Kp_283		*bla*_KPC_	S	S	S	R	S	R	S	S	S	S	
Kp_285		*bla*_KPC_	S	S	S	R	S	R	S	S	S	S	
Kp_673		*bla*_KPC_	S	S	S	S	S	S	S	S	R	S	
Kp_679		*bla*_KPC_	S	S	S	S	S	S	S	S	S	S	
Kp_15	FIIK, FIB KQ, HI1	*bla*_KPC_	R	S	S	S	S	S	R	S	R	S	512
Kp_245	FIIK, FIB KQ, FIB KN, X3	*bla*_KPC_	S	S	S	S	S	R	S	S	S	S	512
Kp_709	FIB KN	*bla*_KPC_	S	S	S	R	S	R	S	S	S	S	101
Kp_9	FIIK, FIB KN	*bla*_KPC_	S	S	S	R	S	R	S	R	S	S	101
Kp_16		*bla*_KPC_	R	S	S	S	S	R	R	S	R	S	
Kp_17		*bla*_KPC_	S	R	R	S	R	S	R	R	R	S	
Kp_284		*bla*_KPC_	S	S	S	S	S	S	S	S	S	S	
Kp_19	FIIK, FIB KN, A/C	*bla*_KPC_	S	R	S	S	R	S	R	R	R	S	101
Kp_49	FIB KQ, FIB KN	*bla*_KPC_	S	S	S	R	S	R	S	S	S	S	101
Kp_640	FIIK, X1,N, M	*bla*_KPC_	S	R	R	S	S	S	R	R	R	S	307
Kp_707	FIB KQ, FIB KN, HI1	*bla*_KPC_	S	S	S	R	R	R	S	R	R	S	307
Ec_136	FIA, FIB, FII	-	S	S	R	S	S	R	R	R	R	S	405
Ec_705	FIB KQ, FIB KN	*bla*_KPC_	S	S	S	R	R	R	R	R	R	S	405
Ec_178	FIIK, FIB KQ, FIA, FIB, I1γ	*bla*_KPC_	S	S	S	R	R	R	R	R	R	S	131

^a^ Kp and Ec are abbreviations for the *K. pneumoniae* and *E. coli strains*, respectively; ^b^ PBRT, PCR-based replicon typing; ^c^ CR, carbapenem-resistance; not detected; ^d^ CN, gentamycin; AK, amikacin; TOB, tobramycin; TZP, piperacillin/tazobactam; FOS, fosfomycin; AMS, ampicillin-sulbactam; CIP, ciprofloxacin; LEV, levofloxacin; CXM, cefuroxime; SXT, co-trimoxazole; ^e^ ST, sequence type.

**Table 2:** Distribution of selected variables associated with the detection of CPE on admission.

Variables	Control	%	Cases	%	*p*
**Age**					
<44	2	4.2%	4	8.3%	NS
45–64	17	35.4%	17	35.4%	NS
>65	29	60.4%	27	56.3%	NS
**Sex**					
Male	30	62.5%	32	66.7%	NS
					
**Origin of Patients**					
Emergency department	40	83.3%	25	52.1%	<0.001
Home	3	6.3%	10	20.8%	NS
^1^ LTCF	-		4	8.3%	0.041
Other hospital	5	10.4%	9	18.8%	NS
Hospital admission during the previous 30 days	11	22.9%	11	22.9%	NS
**Comorbidities**					
Diabetes	3	6.3%	8	16.7%	NS
Renal disease	13	27.1%	7	14.6%	NS
Cardiovascular disease	11	22.9%	6	12.5%	NS
^2^ COPD	7	14.6%	3	6.3%	NS
Cancer	10	20.8%	10	20.8%	NS
Presence of disability	1	2.1%	5	10.4%	NS
Respiratory failure	14	29.2%	9	18.8%	NS
Urinary tract infection	9	18.8%	3	6.3%	NS
Administration of antibiotics	14	29.17%	25	52.08%	0.022

NS: >0.05; ^1^ LTCF, long-term care facility; ^2^ COPD, chronic obstructive pulmonary disease.

**Table 3:** Result of logistic regression analysis to evaluate factors associated with CPE carriage on admission.

Variables	OR	*p* Value	95%CI
Previous use of antibiotics	3.76	0.006	1.45–9.72
Previous hospital admission 30 days	3.00	0.023	1.16–7.71
Emergency department (ED)	0.27	0.010	0.10–0.73

## Data Availability

Data is contained within the article.
